# A Mission Reliability-Driven Manufacturing System Health State Evaluation Method Based on Fusion of Operational Data †

**DOI:** 10.3390/s19030442

**Published:** 2019-01-22

**Authors:** Xiao Han, Zili Wang, Yihai He, Yixiao Zhao, Zhaoxiang Chen, Di Zhou

**Affiliations:** School of Reliability and Systems Engineering, Beihang University, Beijing 100083, China; buaahanxiao@buaa.edu.cn (X.H.); wzl@buaa.edu.cn (Z.W.); zhyixiao@buaa.edu.cn (Y.Z.); buaaczx@buaa.edu.cn (Z.C.); zhoudimail@163.com (D.Z.)

**Keywords:** manufacturing system, health state, operational data, data fusion, mission reliability

## Abstract

The rapid development of complexity and intelligence in manufacturing systems leads to an increase in potential operational risks and therefore requires a more comprehensive system-level health diagnostics approach. Based on the massive multi-source operational data collected by smart sensors, this paper proposes a mission reliability-driven manufacturing system health state evaluation method. Characteristic attributes affecting the mission reliability are monitored and analyzed based on different sensor groups, including the performance state of the manufacturing equipment, the execution state of the production task and the quality state of the manufactured product. The Dempster-Shafer (D-S) evidence theory approach is used to diagnose the health state of the manufacturing system. Results of a case study show that the proposed evaluation method can dynamically and effectively characterize the actual health state of manufacturing systems.

## 1. Introduction

Existing prognostic and health management (PHM) technologies for manufacturing systems are mainly based on the individual performance of production equipment, hardly considering the characterization of the systematic operational health state. With the increasing complexity of modern manufacturing systems, traditional reliability theory is limited in describing complex characteristics of running multi-state manufacturing systems [1], such as multi-state properties and epistemic uncertainties [2]. Reliability modeling of these systems should be extended to characterize the dynamic running state of various components in manufacturing systems. In addition, the massive multi-source heterogeneous operational data generated by manufacturing systems have a significant potential in characterizing the health state of manufacturing systems and accurately solving uncertainties.

Massive operational data in the production process is collected and analyzed to make production decisions for the production management activities, such as production scheduling, maintenance activities, process quality control, based on the actual state of each production element in the manufacturing system. Kammoun [3] combined a data mining approach with the selective maintenance of multi-component manufacturing systems by collecting maintenance data. Shao [4] established a data-driven model for collaborative manufacturing systems considering geometric and physical performances using multi-objective optimization of modified machine settings. Ratava [5] used acceleration sensors to estimate tool and deflect the detection of tool edge’s chipping and small fracture. Wan [6] proposed a manufacturing big data solution for active preventive maintenance on the basis of cloud data processing. Zhang [7,8] studied the quality deviation propagation model of single-station and multi-station assembly parts using stream of variation. Similarly, Chen [9] proposed the QR chain effect of a multi-station manufacturing system using stream of variation. In addition, Ju [10,11] analyzed the evolution of material quality in a multi-station production process. The author proposed a quality flow model to analyze and improve the quality of automotive coating and of the battery production process. Du [12] proposed a cumulative modeling and analysis technique for the quality deviation of multi-station manufacturing systems considering the downstream quality inspection data.

The correlation among these production management activities suggests that considering only a single type of operational data is not enough. For example, taking preventive maintenance in production scheduling is necessary. Product quality fluctuation must be considered in determining the maintenance threshold of manufacturing equipment. Quality management in production processes must also consider the impact of system load reprocessing. Therefore, production management integration is gradually being researched. Liu [13] proposed a reliability evaluation method for hybrid assembly systems by integrating process performance and product quality to optimize maintenance schedules. 

The core of intelligent manufacturing lies in “prediction and manufacturing,” implying the enormous potential of multi-sensor integration [14]. Effective processing of massive, multi-source, and real-time manufacturing data [15] to obtain information related to production management requirements is a basic premise for intelligent analysis and decision making. Data fusion [16] and complex event processes are the main contents of data processing research on the current manufacturing of Internet of Things systems [17]. Wang [18] proposed a tool wear-sensing technique on the basis of multi-sensory data fusion, such as force and vibration, which are used to build an intelligence model for tool condition monitoring. Posada [19] focused on the data fusion of graphics and vision and media technologies, which can enhance the role of operators. Sillitoe [20] proposed a reference design of intelligent signal conditioning and implemented the force and displacement sensors in manufacturing applications. The multi-sensor fusion method [21] is applied for tool condition monitoring in milling. Zhang [22] proposed an overall architecture of big data-based analytics for product lifecycle; the architecture integrates big data analytics and service-driven patterns, thereby helping overcome barriers to clean production. A multi-source information fusion based fault diagnosis [23] methodology is proposed to increase the fault diagnostic accuracy of ground-source heat pump (GSHP) system greatly by using Bayesian network. 

In accordance with above literature review, the state analysis and reliability evaluation of manufacturing components based on data fusion has become a research focus [24]. Cai [25] Proposed a new availability-based engineering resilience metric from the perspective of reliability engineering and developed a corresponding dynamic evaluation methodology based on Bayesian–network. Cai [26] presented a reliability evaluation method based on Bayesian–network, largely simplifying the calculation process effectively. However, existing research still lacks quantitative description indicators for the mission reliability and health state evaluation method of manufacturing systems. These indicators can systematically analyze the overall state and optimize their operational performance [27]. For solving this problem, an operational data fusion method and a health state evaluation method of manufacturing systems are proposed.

The rest of this article is organized in the following manner. Section 2 analyzes the operational data during the production cycle. Section 3 describes the basic definitions of the health state of manufacturing systems. Section 4 presents an evaluation method for the health state model. Section 5 presents a case study of automotive cylinder head production. Section 6 provides the conclusions.

## 2. Health State-Oriented Operational Data Fusion Framework

### 2.1. Big Operational Data Generated in the Production Process

Manufacturing elements, such as humans, equipment, materials, and sensors in manufacturing systems, work together to achieve production requirements. Massive operational data produced in various stages of production, quality control, and equipment maintenance have great value in the analysis and optimization of manufacturing systems.

**Figure 1:**
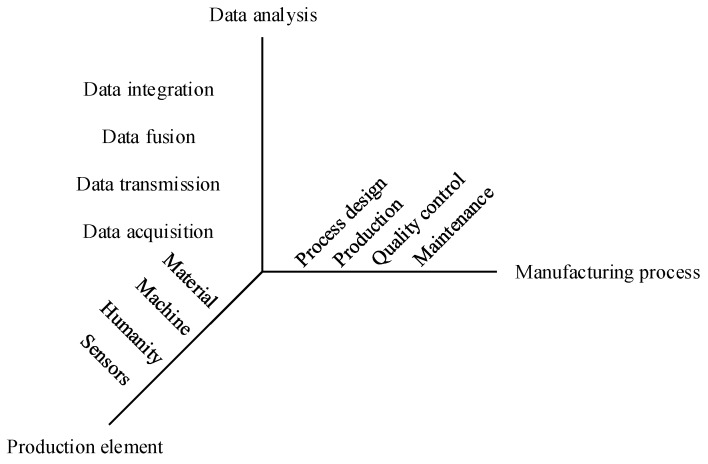
Data sources and applications in production processes.

In the intelligent manufacturing era, multi-source heterogeneous data in different manufacturing stages can be acquired in real time using mass sensors. Manufacturing data can be analyzed to obtain system state information, which then assists in decision making for production activities. At the stage of process design, important process parameters can be obtained based on big operational data to avoid the limitations of traditional parameter design. At the stage of process design, although only the working time and fault records were recorded as equipment data in the past, manufacturing equipment properties can be accurately measured with the development of sensor technology. Dynamic data, such as the wear of each machine tool due to the machining process and the WIP of each production process, including its corresponding machining time, are accurately recorded and analyzed as needed. Moreover, the deviation of the key quality characteristics (KQCs) of WIP can be obtained quickly. The data fusion technology used in the data layer helps eliminate the influence of environmental noise on the data. Furthermore, the performance state of manufacturing equipment can be monitored and predicted on the basis of operation data and PHM technologies. Preventive maintenance can be arranged in consideration of production scheduling and maintenance cost. Manufacturing data usage can be represented by an architecture of “acquisition–transmission–fusion–integration” as Figure 1.

#### 2.1.1. Data Acquisition

Given the distribution and functional characteristics of various production factors in manufacturing systems, such as equipment, humanity, materials, tools, and WIP, various types of sensing devices such as radio frequency identification (RFID), sensors, and ultra-wideband, are deployed on the shop floor. The interconnection and data collection of various production factors ensure reliable real-time acquisition of multi-source information generated by production activities.

#### 2.1.2. Data Transmission

Multi-source heterogeneous data generated by manufacturing systems in production processes, such as equipment operation, quality inspection, and environmental parameters, have different characteristics. These data can be selectively implemented through industrial Ethernet, fieldbus, wireless communication, and other technologies. The effective transmission and exchange of sensory information ensure stable transmission and application of production data on the shop floor.

#### 2.1.3. Data Fusion

Manufacturing plants have complex environmental and production characteristics that result in redundancy, out-of-order conditions, and strong uncertainty for operational data. Therefore, verification, smoothing, and filtering of manufacturing data with large capacity and low value density can extract effective information from massive operational data. This extraction also includes data fusion for multi-sensor monitoring data.

#### 2.1.4. Data Integration

Operational data are perceived and then applied for shop floor management and production process optimization to increase the accuracy of dynamic production scheduling, quality control, and predictive maintenance. Enterprise resource planning can be further integrated to enable the transparent monitoring and precise optimization of manufacturing systems. 

### 2.2. Operational Data Fusion Model in Production Processes

**Figure 2:**
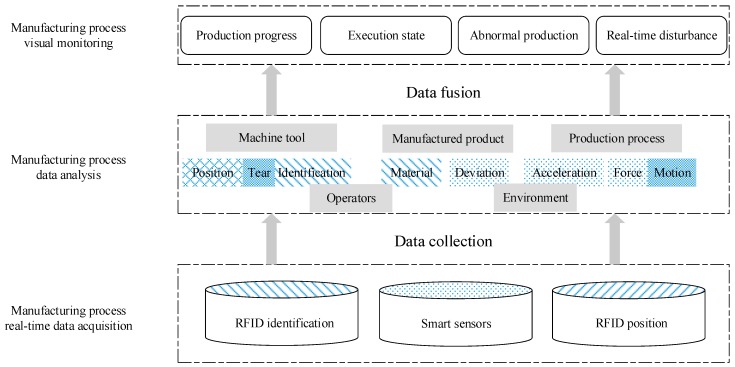
Operational data fusion structure.

Manufacturing workshops have complex environments with various production modes, which lead to large manufacturing data capacity and low value density. Manufacturing workshop must also be transformed into production and management applications through data filtering, fusion, classification, correlation, and other processing operations. Effective data and intelligent analysis methods are used to achieve value-added applications of massive operational data. Figure 2 illustrates the operational data fusion structure of an entire manufacturing system.

The different shading in Figure 2 represents different data of RFID identification, smart sensors and RFID position. The shading in the data analysis blocks represents the single or multiple data types required to extract the data features.

Manufacturing element identification and sensing technology are the basis for realizing the interconnection of people, machines, objects, and information systems in a manufacturing workshop. Manufacturing elements are identified through various types of sensing devices, manufacturing events are realized, and real-time data are generated in the physical workshop. Identification, sensing, and positioning technologies are the main sources of data acquisition.

Object identification technology based on RFID is increasingly becoming mature in the manufacturing industry. It can be used to identify, collect, monitor, and manage many types of manufacturing elements, such as workshop personnel, materials, work-in-progress, and cutting tools. Discrete manufacturing can also be clearly understood. The automatic identification of parts in the process and the real-time synchronization of logistics and information flow effectively improve the traceability and controllability of production processes. Sensing technology mainly collects various parameters of equipment, products, tools, and other sensing objects in the workshop through various sensors, including equipment operation, quality inspection, workshop environment, and other data. Real-time positioning technology is used to locate, track, and monitor multiple types of manufacturing resources in discrete workshops. This technology plays a critical role in the intelligent improvement of workshops.

Given the collected operational data, operational states of production processes can be analyzed by data fusion. Original data can be fused to eliminate the uncertainty and volatility introduced by the environment and sensor performance. This fusion can improve data reliability. Then, feature extraction is performed on the processed data, such as the edge, direction, and speed of the target. Last, feature information is comprehensively analyzed and processed. For complex states, such as tool wear, comprehensive analysis can be performed through multi-sensor information to obtain results such as acceleration and displacement sensors.

Different types of information can be collected by primary processing and analysis and can be employed for different production management activities, such as production scheduling and maintenance time. In the directional screening process of information, certain valuable data and information are eliminated or neglected. Moreover, they can hardly support the health state diagnosis of manufacturing systems in the existing evaluation model. Therefore, a new manufacturing system health state model must be established to maximize the vast amount of operational data available and thus obtain an accurate health state system.

## 3. Basics of Health State Modeling for Manufacturing Systems

### 3.1. Multi-State Connotation of Manufacturing Systems

As working time increase, the production efficiency and production quality of manufacturing systems can have a significant downward trend. Therefore, manufacturing systems have a typical polymorphism, especially in the flexible manufacturing pattern with the characteristics of multi-variety and small lots. Considering formation mechanism, manufacturing system polymorphism can be divided into active/deterministic polymorphism and passive/random polymorphism.

#### 3.1.1. Active/Deterministic Polymorphism

Failure rate of manufacturing equipment is related to operating time and task loads. Therefore, manufacturing equipment will reflect different performance states in different production scheduling, especially for those consist of multiple manufacturing units. When a processing unit fails, the manufacturing equipment loses certain functions, but the failure may not affect the execution of other functions. Manufacturing system polymorphism caused by the production scheduling is defined as active/deterministic polymorphism and can be pre-estimated.

#### 3.1.2. Passive/Random Polymorphism

The function manufacturing systems is to produce qualified products. However, as the production process progresses, the performance state of equipment units shows an irreversible trend of degradation because of sectors such as wear and fatigue. Accumulating this degradation may not directly lead to a failure in the production process. However, the overall production quality may be degraded until the unqualified product appears. Therefore, performance state of manufacturing equipment is also reflected in the quality state of the manufactured product. Manufacturing system polymorphism, which is caused by the degradation of performance and affects the quality state of WIP, is defined as passive polymorphism.

### 3.2. Mission Reliability Modeling for Manufacturing Systems

The valuable outputs of manufacturing systems is the final manufactured products with limited makespan and production quality. For the reliability model of production task-oriented manufacturing systems, considering the impact of multiple task requirements on system reliability is necessary. Considering the previous discussion, we can conclude that sensor technology development in intelligent manufacturing provides enough operational data for multi-tasking reliability evaluation of manufacturing systems with multiple production tasks, especially the real-time multi-source data in the shop floor. Therefore, manufacturing system reliability changes with the performance degradation of manufacturing equipment and dynamic scheduling, including workload adjustment and system constitution. The following are the variations of manufacturing system reliability considering dynamic production tasks.

**Figure 3:**
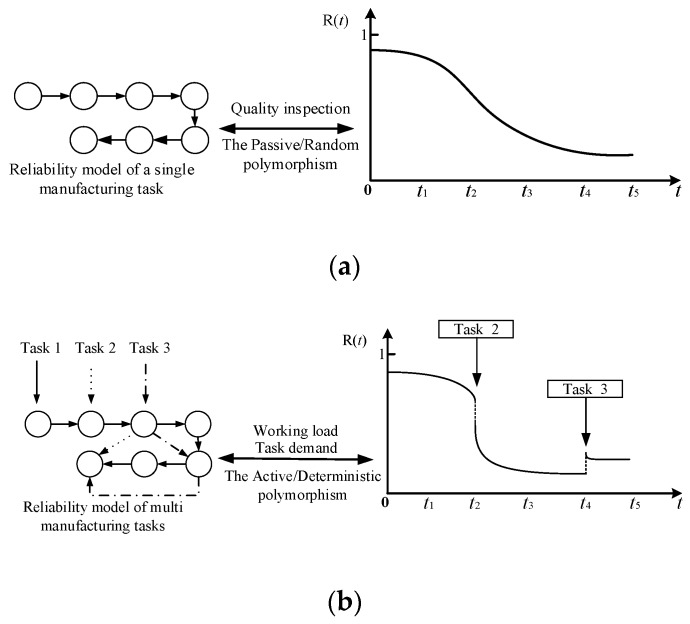
Reliability variation trends of manufacturing systems: (**a**) Basic reliability of manufacturing systems; (**b**) Mission reliability of manufacturing systems.

Figure 3 displays that the mission reliability of manufacturing systems is closely related to the performance state of manufacturing equipment, execution state of production tasks, and quality state of manufactured products. The three jointly determine the possibility that manufacturing systems can complete the specified production tasks with time and resource constraints. Moreover, the health state of manufacturing systems that characterizes the ability to perform functions is related to the three elements. The performance state of manufacturing equipment is the basis of the health state of manufacturing systems. Performance state also expects the decline of health state. The quality state of manufactured products is determined by the performance state of corresponding manufacturing equipment, which is a direct reflection of the health state of manufacturing systems. The execution state of production tasks focuses on the dynamic state changes of manufacturing systems under different mission profiles.

## 4. Health State Evaluation Method for Manufacturing Systems

### 4.1. Mission Reliability Oriented Health State Evaluation Framework

**Figure 4:**
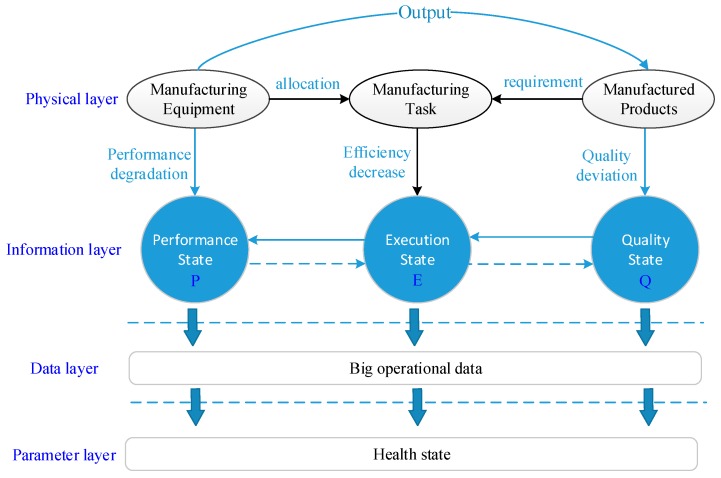
Health state evaluation framework of manufacturing systems.

The health state of manufacturing systems depends on the performance state of manufacturing equipment. Their health state is also reflected in the quality state of manufactured products. Manufacturing companies perceive that customer demands, such as makespan, are also important indicators when evaluating the health state of manufacturing systems. To accurately reflect their health state, the mission reliability of manufacturing systems is defined as the ability to continuously output products that meet production requirements under a certain profile of a production task. The operational data generated during the production process can be used to model and analyze the mission reliability of manufacturing systems from the three aspects, as shown in the following Figure 4.

### 4.2. Quantify the Performance State of Manufacturing Equipment

Manufacturing equipment mainly processes WIP by using equipment components, such as tools. Tool wear is one of the most typical degradation phenomena in metal cutting. The main monitoring signals of the current tool wear state detection are cutting force signal, vibration signal, cutting temperature signal, acoustic emission signal, and acceleration signal. The wear state information of the tool is extracted by performing noise reduction processing on the observation signal during the cutting process. For multi-sensor monitoring signals, data fusion at the data level is first required to collect accurate information. Furthermore, data fusion at the feature level is performed on different signals to obtain a degradation state of manufacturing components.

Current information on machine tool spindle can be used to evaluate the performance state of machine tools. The output torque of spindle motors can be expressed as follows:

(1) $$T_{c}=J\frac{d\omega }{dt}+B\omega +T_{t}.$$

Despite the loss of rotor winding, spindle motor output torque $T_{e}$ can be calculated as follows:

(2) $$T_{e}=K_{e}I_{e},$$

(3) $$I_{e}=\sqrt{i_{ab}^{}{}^{2}+i_{ac}^{}{}^{2}+i_{bc}^{}{}^{2}}.$$

The total disturbance torque of the spindle motor can be expressed as:

(4) $$T_{t}=T_{a}+T_{fco}+\delta T_{f}+T_{c},$$

where $T_{a}$ represents the torque caused by the failure of spindle mechanical components.

When the spindle rotates at a constant speed without load, the value of $T_{c}$, $\delta T_{f}$, $J\frac{d\omega }{dt}$ are 0. Therefore:

(5) $$T_{a}=K_{e}I_{e}-B\omega -T_{f}.$$

If the value of *Bω* and *T_t_* are constant, then the output torque can change as mechanical components fail. Assuming that current threshold $I_{e}^{\prime }$ corresponds to the fault and ideal value $I_{0}$ corresponds to the perfect performance state of a new spindle, performance state *P* of machine tools can be defined as follows:

(6) $$P=\frac{I_{e}^{\prime }-I_{e}}{I_{e}^{\prime }-I_{0}}.$$

### 4.3. Quantify the Execution State of Production Tasks

The execution state of production tasks describes the ability of manufacturing systems to produce the specified number of products on schedule. As the performance state of the manufacturing equipment degrades, the processing time of subtasks corresponding to the equipment can be prolonged. In addition, maintenance time may be introduced because of the failure caused by the performance degradation of the equipment. Therefore, the execution state of production tasks is evaluated according to the probability that the makespan of production processes is lower than the required period demand.

The processing time of the equipment for the same production tasks can increase as the service time increases. Thus, the recorded service time of the manufacturing equipment can be used to judge equipment state degradation. RFID can be employed to determine how long WIP passes through the manufacturing equipment. Such data can be stored and extracted for analysis during the reliability assessment of manufacturing system tasks. All the manufacturing equipment in a degraded state can be identified with the performance degradation evaluation method in the previous section. The corresponding subtask completion time may be prolonged to varying degrees compared with the expected completion time in the production scheduling. Age factor α is introduced to characterize the impact of equipment age on completion time and can be evaluated by the historical data from the corresponding manufacturing equipment using RFID. Therefore, the processing time of subtask Pi is as follows:

(7) $$t_{Pi}=\alpha_{Pi}{}_{Mj}{\tilde{t}}_{Pi}.$$

when the equipment fails and requires passive shutdown maintenance, maintenance time leads to an increase in the makespan. Moreover, if the equipment is in a degraded state, then the required maintenance time increases as time passes. Such an increase can be expressed as follows:

(8) $$t_{Mj}={\tilde{t}}_{Mj}+\beta \sum_{i=1}^{k-1}t_{ij}.$$

The following is the total completion time for manufacturing systems with *n* degraded manufacturing equipment:

(9) $$T=\sum_{j=1}^{n}t_{Mj}\times \left[1-R_{Mj}\right].$$

The execution state of production tasks can be expressed as follows:

(10) $$E=\mathrm{Pr}(T\leq T_{0}).$$

### 4.4. Quantify the Quality State of WIP

A process model can be established to represent the deviation $Y_{k}(t)$ of the quality characteristics *k* of a product:

(11) $$Y_{k}(t)=\varphi_{k}+a_{k}^{T}V(t)+b_{k}^{T}Z(t)+V{\left(t\right)}^{T}A_{k}Z(t).$$

$\varphi_{k}$ is the baseline constant, and the $a_{k}^{T}$ and $b_{k}^{T}$ represent the linear effects of controllable process variables and environmental noise variables, respectively, on product quality deviations. Both can be obtained through engineering practice by employing a specific physical process model.

Then, $q_{k}$ is defined as the key quality deviation indicator of manufactured products to characterize quality deviation:

(12) $$\begin{array}{ll} q_{k}\left(t\right) & =E\left[E\left(Y_{k}{\left(t\right)}^{2}|V\left(t\right)\right)\right]\\ & =E\left[V{\left(t\right)}^{T}\right]\Phi E\left[V\left(t\right)\right]+\psi^{T}E\left[V\left(t\right)\right]+\sum_{i=1}^{n}\phi_{ii}Var\left[V\left(t\right)\right]+\Theta \end{array}$$

(13) $$\Phi =A_{k}\mathrm{cov}\left(Z(t)\right)A_{k}^{T}+a_{k}a_{k}^{T}$$

(14) $$\psi^{T}=2\left(b_{k}^{T}\left(Z(t)\right)A_{k}^{T}+\varphi_{k}a_{k}^{T}\right)$$

(15) $$\Theta =b_{k}^{T}\mathrm{cov}\left(Z(t)\right)b_{k}+\varphi_{k}{}^{2}$$

The quality qualification of KQC k can be simplified and expressed as the following formula on the basis of Gamma distribution characteristics:

(16) $$q_{k}\left(t\right)=\upsilon^{T}\Phi \upsilon t^{2}+\psi^{T}\upsilon t+\sum_{i=1}^{n}\phi_{ii}\upsilon_{i}/\theta_{i}{}^{2}t+\Theta$$

(17) $$Q_{k}(t)=\{\begin{array}{ll} 0 & q_{k}\left(t\right)>a_{k}\;\\ 1-\frac{q_{k}\left(t\right)}{a_{k}} & q_{k}\left(t\right)\leq a_{k} \end{array}$$

(18) $$a_{k}={\left[\frac{6C_{pm}}{USL-LSL}\right]}^{2}$$

### 4.5. Evaluate Health State of Manufacturing Systems on the Basis of D-S Evidence Theory

D-S evidence theory was introduced by Dempster and then developed by Shafer. The theory is widely applied to data fusion in production processes because of its outstanding performance in uncertainty models and processes. 

**Figure 5:**
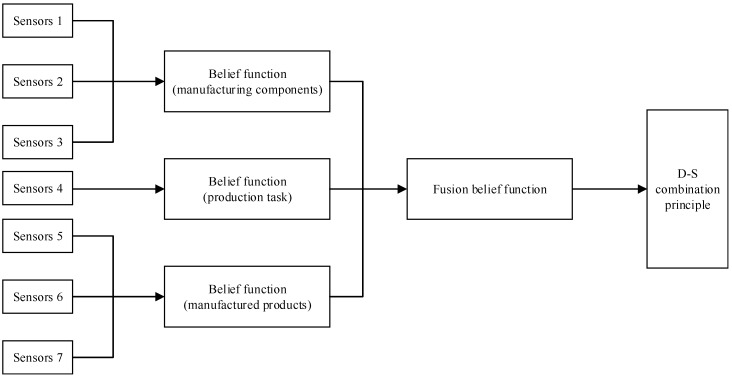
Fusion diagnosis process of manufacturing systems.

The health state of manufacturing systems is divided into three levels—a high performance state, a medium state, and a low state. The three aspects of data fusion—degradation of manufacturing components, increase in processing time, and degradation of the quality deviation of manufactured products—are the evident sources for the final decision making of manufacturing equipment’s performance state. The final performance state category is determined by the fusion diagnosis process as shown in Figure 5.

In this study, we require fusing the information of risk factors with D-S evidence theory. However, evidence of risk factors may conflict with each other. Therefore, we define three linguistic variables—L (low), M (medium), and H (high)—to express the basic characteristic of health state.

- Step 1. Constructing membership function

Define $\mu_{L}(k)$, $\mu_{M}(k)$, and $\mu_{H}(k)$ as the membership functions of L, M, and H, where trapezoid subordinate function is used to simulate the restrictions:

(19) $$\mu_{L}(k)=\{\begin{array}{cc} 1, & k\leq 0.1\\ \frac{0.9-k}{0.9-0.1}, & 0.1<k\leq 0.9\\ 0, & k>0.9 \end{array}$$

(20) $$\mu_{M}(k)=\{\begin{array}{cc} 0, & k\leq 0.1\\ \frac{k-0.1}{0.4-0.1}, & 0.1<k\leq 0.4\\ 1, & 0.4<k\leq 0.6\\ \frac{0.9-k}{0.9-0.6}, & 0.6<k\leq 0.9\\ 0, & k>0.9 \end{array}$$

(21) $$\mu_{H}(k)=\{\begin{array}{cc} 0, & k\leq 0.1\\ \frac{k-0.1}{0.9-0.1}, & 0.1<k\leq 0.9\\ 1, & k>0.9 \end{array}$$

- Step 2. Determining the state level of production tasks and generating BPA
  (22) $$\left\{ \begin{array}{l} \mu_{i}(P)\times P=\nu_{i}\\ \mu_{i}(E)\times E=\nu_{i}\\ \mu_{i}(Q)\times Q=\nu_{i} \end{array}\right.$$

Basic probability assignment (BPA) is an important component in D-S evidence theory. In data fusion application systems based this theory, BPA should be given and fused by Dempster’s combination rule. However, determining BPA is still an open issue. Many authors have addressed this problem using different approaches. In this section, a method of turning FMV to a set of BPAs is proposed.

BPA generation method suggests that the higher the mass of a risk level (L, M, or H), the more they can be aggregated in the combination process. Thus, this method has a high convergence rate in extracting the main risk feature.

For example, if the vector of $Q_{i}$ is (0.1, 0.7, 0.2), the BPAs of $Q_{i}$ are m(M)=0.7,m(M,H)=0.2,m(L,M,H)=0.1.

- Step 3. Obtaining the health state by evidence combination

After obtaining all related BPAs generated through the three belief vectors, Dempster’s combination rule must be employed to aggregate them, which is formulated as follows:

(23) $$m\left(A\right)=\{\begin{array}{c} 0,A=\emptyset \\ \frac{1}{1-k}\sum_{B\cap C=A}m_{1}\left(B\right)m_{2}\left(C\right),A\neq \emptyset ,k=\sum_{B\cap C=\emptyset }m_{1}\left(B\right)m_{2}\left(C\right) \end{array}.$$

Pignistic probability transformation is also involved. It equally allocates BPA to each subset in the aggregation process, which is shown as follows:

(24) $$BetP\left(\left\{x\right\}\right)=\sum_{x\in A\subseteq \Theta }\frac{1}{\left|A\right|}\frac{m\left(A\right)}{1-m\left(\emptyset \right)},m\left(\emptyset \right)\neq 1,$$

where |A| denotes the element numbers in set A.

## 5. Case Study

### 5.1. Background

The cylinder head is an important component of engines with several apertures, which play an important role in supporting engine structural stability and determining assembly standards. The strict requirements on the surface roughness, geometry, processing size, and positional accuracy of cylinder head indicate that the corresponding processes are complex. The manufacturing quality of cylinder head directly affects the assembly accuracy of engines and the reliability of the use stage. Therefore, accurate and effective health state modeling and analysis of the cylinder head manufacturing system can provide an effective assessment production decisions and maintenance activity, ensuring that manufacturing systems can continuously produce high-quality products and reduce quality loss.

KQC in the production process of cylinder head is determined by analyzing the typical manufacturing defects and corresponding production process of unqualified cylinder head, as listed in Table 1.

### 5.2. Numerical Example

The manufacturing equipment M_1,_ M_2,_ M_3,_ and the corresponding manufacturing elements constitute a subsystem. To evaluate the health state of manufacturing systems, the generated operation data in the production process are collected, which can characterize the performance state of manufacturing equipment, the execution state of production tasks, and the quality state of products.

#### 5.2.1. Performance State of the Manufacturing Equipment

The performance state of CNC machine tools can be evaluated by the spindle current. Firstly, the machine current is measured under different working conditions. Then, compare the value with the current amplitude corresponding to the wear-free state and the most serious acceptable wear state. Finally, the performance state of the manufacturing equipment can be obtained, as shown in the Table 2.

#### 5.2.2. Quantify the Execution State of the Production Task

According to the statistical data of equipment failure, the failure rate of manufacturing equipment is modeled without consideration of maintenance activities. Given the wide application of Weibull distribution in the failure rate modeling of electromechanical systems, the following expressions can be obtained:

$$\lambda_{1}(t)=1.11^{0.00053t}\times 2.4\times 10^{-5}t^{2},$$

$$\lambda_{2}(t)=1.12^{0.00079t}\times 3.91\times 10^{-5}t^{2},$$

$$\lambda_{3}(t)=1.10^{0.00125t}\times 2.89\times 10^{-5}t^{2}.$$

Therefore, the expected delay for the three manufacturing equipment is as follows:

$$T=\sum_{j=1}^{n}t_{Mj}\times \left\{1-\exp\left[-\int_{0}^{t}\lambda_{Mj}(t)\mathrm{dt}\right]\right\}.$$

Since the repair time of the manufacturing equipment in this subsystem is higher than the acceptable delay time, execution state of production tasks can be equivalent to the reliability of the manufacturing equipment during the makespan:

$$E=\mathrm{Pr}(T\leq T_{0})=R_{Mj}=\exp\left[-\int_{0}^{t}\lambda_{Mj}(t)\mathrm{dt}\right]$$

The execution state of production tasks are:

$$E_{M_{1}}(50)=0.367;E_{M_{2}}(50)=0.758;E_{M_{3}}(50)=0.298.$$

#### 5.2.3. Quality State of WIP

Among the three manufacturing equipment, three KQCs correspondingly exist—the concentricity of camshaft hole, the diameter of rocker shaft hole, and the flatness of the underside of the cylinder head. The three deviations are denoted by $Y_{1}(t)$, $Y_{2}(t)$, $Y_{3}(t)$, respectively. Process control variables that cause the three kinds of deviations are the radial runout $V_{1}\left(t\right)$, radial direction agitation $V_{2}\left(t\right)$, and axial agitation $V_{3}\left(t\right)$ of the reamer, respectively. Equipment vibration affects the concentricity and diameter of the guide hole. Moreover, equipment vibration is considered a noise variable and denoted by *Z*(*t*). 

Among the above three manufacturing equipment, there are three key quality characteristics correspondingly, which are the concentricity of camshaft hole, the diameter of rocker shaft hole, and the flatness of the underside of the cylinder head. The three deviations are denoted by $Y_{1}(t)$, $Y_{2}(t)$, $Y_{3}(t)$ respectively. The process control variables that cause the above three kinds of deviations are the radial runout $V_{1}\left(t\right)$, radial direction agitation $V_{2}\left(t\right)$, and axial agitation $V_{3}\left(t\right)$ of the reamer respectively. The vibration of the equipment will affect the concentricity of the diameter and the guide hole, and it is considered as a noise variable and denoted by Z(t).

The process model can be established, and the results are as follows:

$$Y_{1}(t)=0.774V_{1}(t)+0.363Z(t)-0.0581V_{1}(t)Z(t),$$

$$Y_{2}(t)=0.572V_{2}(t)+0.213Z(t)+0.0427V_{2}(t)Z(t),$$

$$Y_{3}(t)=0.682V_{3}(t)+0.323Z(t)+0.0432V_{3}(t)Z(t).$$

The KQCs of products corresponding to the three key processes under investigation can be obtained using Equation (17), and the details are as follows:

$$q_{1}\left(t\right)\;=1.26\times 10^{-8}t^{2}+2.38\times 10^{-5}t+1.32\times 10^{-5},$$

$$q_{2}\left(t\right)\;=1.35\times 10^{-8}t^{2}+1.71\times 10^{-5}t+4.54\times 10^{-6},$$

$$q_{3}\left(t\right)\;=2.25\times 10^{-8}t^{2}+1.8\times 10^{-5}t+1.04\times 10^{-5}.$$

Furthermore, the fluctuation threshold of each quality deviation can be determined as follows:

*a*_1_ = 0.0025, *a*_2_ = 0.0015, *a*_3_ = 0.0018.

When *t* = 50, the quality state of manufactured products is as follows:

$$Q_{M_{1}}(50)=0.506,\;Q_{M_{2}}(50)=0.404,\;Q_{M_{3}}(50)=0.463$$

#### 5.2.4. Health State of Manufacturing Equipment Based on D-S Evidence Theory

After obtaining the three operational states at time *t* through the given steps, the weight of each state value $\mu_{k}\left(S\right)$ can be calculated using the trapezoidal membership function in Equations (19)–(21). By employing Equation (22), the following elements in the belief structure of *P* can be obtained:

$$v_{1}=\sum_{i}\mu_{1}\left(S_{i}\right)\mu_{\tilde{P}}\left(S_{i}\right)=\left[0.75,0.50,0.57\right]{\left[0.1875,0.5,0.4125\right]}^{T}=0.62575$$

$$v_{2}=\sum_{i}\mu_{2}\left(S_{i}\right)\mu_{\tilde{P}}\left(S_{i}\right)=\left[0.75,0.50,0.57\right]{\left[0.5,1,1\right]}^{T}=1.445$$

$$v_{3}=\sum_{i}\mu_{3}\left(S_{i}\right)\mu_{\tilde{P}}\left(S_{i}\right)=\left[0.75,0.50,0.57\right]{\left[0.8125,0.5,0.5875\right]}^{T}=1.19425$$

Therefore, the belief structures of *P*, *E*, and *Q* are $V_{\tilde{P}}=\left(0.192,0.442,0.366\right)$, $V_{\tilde{E}}=\left(0.262,0.383,0.355\right)$, and $V_{\tilde{Q}}=\left(0.274,0.5,0.226\right)$, respectively. Considering the law of BPA generation that the higher the mass of a state level, the more it can be aggregated in this combination process, Table 3 lists the BPAs of the three operational states generated by the given belief structures. 

The following are the aggregation results:

$$\begin{array}{c} m_{P⊕E⊕Q}\left(L\right)=0,m_{P⊕E⊕Q}\left(M\right)=0.9086,m_{P⊕E⊕Q}\left(H\right)=0,\\ m_{P⊕E⊕Q}\left(L,M\right)=0.0137,m_{P⊕E⊕Q}\left(M,H\right)=0.0664,m_{P⊕E⊕Q}\left(L,M,H\right)=0.0113 \end{array}$$

Thus, the health state-containing machine performance, task execution, and WIP quality for manufacturing systems at time t are concluded as follows:

P(L)=0.0106,P(M)=0.9524,P(H)=0.0370,

implying that health state should be subjectively deemed to be a Medium level.

### 5.3. Sensitivity Analysis and Comparative Study

From the above discussion, it is clear to make a subjective judgment that the current health state of cylinder head manufacturing system is in a medium level, which means that the system does not require preventive maintenance activities. It is worth noting that the performance state of the manufacturing equipment gradually decreases over time, while the other two states of manufacturing system do not change significantly for a given production scheduling. Therefore, we record the current amplitude of the manufacturing equipment within the scope of the production plan and calculate the real-time dynamic health states of the system, the final results are shown in the following Table 4.

The health state of the manufacturing system is significantly degrading as the components of the manufacturing equipment degrade. However, the evaluation of final health state takes both the execution state of production tasks and quality state of manufactured products into account. Production decisions could be made through system-level data and component-level data, that is, the entire system is in a good condition, and the manufacturing equipment M_1_ needs preventive maintenance.

Furtherly, the system health state are compared with different characteristics attributes, as shown in Table 5. From the perspective of equipment shutdown, the execution states of the equipment M1 and M3 are poor, and it is necessary to consider scheduling maintenance activities. However, the performance states and manufactured products quality states of the both are in an acceptable position, resulting in the manufacturing system being in an intermediate health state. The actual production results also show that the manufacturing system can maintain the required production capacity during the production cycle, and the quality consistency of the manufactured products meets the requirements.

It can also be seen from Table 2, Table 3, Table 4 and Table 5 that the data fusion of the decision-making layer can reduce the influence of the uncertainty of state recognition and improve the accuracy of the diagnosis for the health state of the manufacturing system.

## 6. Conclusions

This study proposes a mission reliability-driven manufacturing system health state evaluation method based on fusion of operational data, integrating the performance state of manufacturing equipment, the execution state of production tasks and the quality state of manufactured products from the perspective of completing production tasks on demand. On this basis, an operational data fusion framework for manufacturing system is proposed, which fully utilizes the multi-source heterogeneous data in the production process to comprehensively and dynamically evaluate the health state of the manufacturing systems. The current amplitude monitoring data of the machine tool spindle of the manufacturing equipment is used to identify tool wear and dynamically evaluate the performance state of the manufacturing equipment. Since the delay caused by equipment downtime is considered to be a major factor affecting the production schedule, the failure rate data of the manufacturing equipment is used to estimate the execution state of the production task. Then, establish a process model of the quality characteristic deviation and estimate the quality state of the manufactured products. In addition, D-S evidence theory is used for the final data fusion to obtain the health state of the manufacturing systems. Finally, a case study of the cylinder head manufacturing system is carried out to verify the effectiveness of the method. The results show that the uncertainty of state recognition for a single model has the risk of false negatives and false alarms. The health state of the manufacturing system needs to be analyzed and identified from the system level, not just the degradation of components or the failure rate of equipment. In addition, the proposed evaluation method can also monitor the quality state of the manufactured products and enhance the sustainable competitiveness of the manufacturers. 

Future research on the health state evaluation method of the manufacturing systems will focus on the following points:

(1) Enrich the performance status monitoring parameters of different types of manufacturing equipment
(2) Uncertainty in manufacturing system health state identification
(3) Exploring the impact of manufacturing system health status on production activities and maintenance activity decisions

## Figures and Tables

**Table 1:** Machining process of the cylinder head of KQCs.

Process ID Number	KQC	Processing Procedure	Manufacturing Equipment
1	Concentricity Diameter	Processing conduit hole	M_1_
2	Concentricity	Fine boring of camshaft hole	M_2_
3	Aperture accuracy	Fine boring of rocker shaft hole	M_3_

**Table 2:** Performance state of the manufacturing equipment.

	M_1_	M_2_	M_3_
Current amplitude without wear	18.62	16.83	17.05
Current amplitude under the most serious acceptable wear state	18.70	16.89	17.12
Current amplitude	18.64	16.86	17.08
Performance state	0.75	0.50	0.57

**Table 3:** BPAs of health state factors.

P	E	Q
m(M)=0.442	m(M)=0.383	m(M)=0.5
m(M,H)=0.366	m(M,H)=0.355	m(L,M)=0.274
m(L,M,H)=0.192	m(L,M,H)=0.262	m(L,M,H)=0.226

**Table 4:** Performance state of the manufacturing equipment.

	M_1_	M_2_	M_3_	P(L)	P(M)	P(H)
Current amplitude without wear	18.62	16.83	17.05	/	/	/
Current amplitude under the most serious acceptable wear state	18.70	16.89	17.12	/	/	/
Current amplitude (*t* = 0)	18.64	16.86	17.08	0.0106	0.9524	0.0370
Current amplitude (*t* = 5)	18.64	16.85	17.08	0.0110	0.9528	0.0362
Current amplitude (*t* = 10)	18.64	16.86	17.08	0.0326	0.9330	0.0344
Current amplitude (*t* = 15)	18.65	16.86	17.08	0.0718	0.8954	0.0328
Current amplitude (*t* = 20)	18.65	16.86	17.08	0.1025	0.8674	0.0301
Current amplitude (*t* = 25)	18.66	16.87	17.08	0.1433	0.8281	0.0286
Current amplitude (*t* = 30)	18.66	16.86	17.08	0.1865	0.7872	0.0263
Current amplitude (*t* = 35)	18.66	16.87	17.09	0.2157	0.7604	0.0239
Current amplitude (*t* = 40)	18.67	16.87	17.08	0.2496	0.7300	0.0204
Current amplitude (*t* = 45)	18.67	16.87	17.08	0.2741	0.7082	0.0177
Current amplitude (*t* = 50)	18.68	16.87	17.08	0.3024	0.6817	0.0159

**Table 5:** Comparison between system health state and different characteristics attributes.

	M_1_	M_2_	M_3_
Performance state	0.75	0.50	0.57
Execution state	0.367	0.758	0.298
Quality state	0.506	0.404	0.463
System health state	P(L)=0.0106	P(M)=0.9524	P(H)=0.0370
